# Involvement of transcribed lncRNA uc.291 in hyperproliferative skin disorders

**DOI:** 10.1186/s13062-023-00435-0

**Published:** 2023-12-01

**Authors:** Mara Mancini, Simone Sergio, Angela Cappello, Timea Farkas, Francesca Bernassola, Claudia Scarponi, Cristina Albanesi, Gerry Melino, Eleonora Candi

**Affiliations:** 1 Istituto Dermopatico Dell’Immacolata, IDI-IRCCS, 00167 Rome, Italy; 2 Department of Experimental Medicine, University of Rome “Tor Vergata”, 00133 Rome, Italy; 3 Interdisciplinary Department of Medicine, University of Bari “Aldo Moro”, 70121 Bari, Italy

**Keywords:** Epidermis, Psoriasis, Hyperproliferative skin disorders, De-differentiation, lncRNA, ACTL6A

## Abstract

The uc.291 transcript controls keratinocytes differentiation by physical interaction with ACTL6A and subsequent induction of transcription of the genes belonging to the epidermal differentiation complex (EDC). Uc.291 is also implicated in the dedifferentiation phenotype seen in poorly differentiated cutaneous squamous cell carcinomas. Here, we would like to investigate the contribution of uc.291 to the unbalanced differentiation state of keratinocytes observed in hyperproliferative skin disorders, e. g., psoriasis. Psoriasis is a multifactorial inflammatory disease, caused by alteration of keratinocytes homeostasis. The imbalanced differentiation state, triggered by the infiltration of immune cells, represents one of the events responsible for this pathology. In the present work, we explore the role of uc.291 and its interactor ACTL6A in psoriasis skin, using quantitative real-time PCR (RT-qPCR), immunohistochemistry and bioinformatic analysis of publicly available datasets. Our data suggest that the expression of the uc.291 and of EDC genes loricrin and filaggrin (*LOR, FLG)* is reduced in lesional skin compared to nonlesional skin of psoriatic patients; conversely, the mRNA and protein level of ACTL6A are up-regulated. Furthermore, we provide evidence that the expression of uc.291, *FLG* and *LOR* is reduced, while *ACTL6A* mRNA is up-regulated, in an in vitro psoriasis-like model obtained by treating differentiated keratinocytes with interleukin 22 (IL-22). Furthermore, analysis of a publicly available dataset of human epidermal keratinocytes treated with IL-22 (GSE7216) confirmed our in vitro results. Taken together, our data reveal a novel role of uc.291 and its functional axis with ACTL6A in psoriasis disorder and a proof of concept that biological inhibition of this molecular axis could have a potential pharmacological effect against psoriasis and, in general, in skin diseases with a suppressed differentiation programme.

**Supplementary Information:**

The online version contains supplementary material available at 10.1186/s13062-023-00435-0.

## Introduction

The human skin is a physical and immune barrier whose essential function is to protect the body against water loss, mechanical insults, and pathogen infection. This tissue has an active metabolic and renewing profile and is composed of two distinct compartments: the dermis and the epidermis [1, 2]. The dermis is the innermost, fibrous layer supporting tissue architecture, vasculature, and innervation; within the dermis reside various types of immune cells, which give the skin the ability to serve as an immune organ [3]. Given its fundamental role in protecting the organism under so many physiological aspects, the skin must be subject to very refined metabolic and/or gene transcriptional regulation to maintain its homeostasis [4, 5]. However, many problems can occur during skin regeneration/renewal, especially at the immune level, leading to skin disorders such as skin cancer and inflammation-mediated skin diseases, such as lichen planus, atopic dermatitis, and psoriasis, collectively known as immune-mediated skin inflammatory diseases (sIMIDs) [6–10]. Among them, psoriasis is an immune-mediated skin disorder that affects around 2% of the population worldwide, although its incidence and prevalence vary between different countries [11]. In addition, this pathology is often associated with a higher risk of cardiovascular diseases and comorbidities, such as obesity, metabolic syndrome, diabetes, arthritis, and others [12, 13]. Clinicians identified various subtypes of psoriasis, including guttate, pustular, and erythrodermic [11]. However, the most common and well-recognised morphological presentation of psoriasis is plaque-type psoriasis. The disease is characterised by the formation of demarked erythematous plaques. Scales are the result of a hyperproliferative epidermis with premature maturation of keratinocytes and incomplete cornification, with retention of nuclei in the stratum corneum (parakeratosis). In skin plaques, impaired keratinocyte differentiation is also observed in the upper layers and massive immune cell infiltration in the dermis [14, 15].

The appearance of psoriasis is the result of aberrant crosstalk between epidermal keratinocytes and immune cells that reside within the skin. Regarding the aetiology of the disease, its appearance is mainly due to environmental stimuli, including stress, UV irradiation, exposure to autoantigens, Streptococcal infections, which occur in genetically susceptible individuals [16, 17]. In fact, several genomic loci have been identified as the cause of genetic susceptibility to psoriasis [18]. In recent years, besides the contribution of genetic and environmental components to the pathogenesis of psoriasis, some evidence identified the role of epigenetic mechanisms, and those exerted by noncoding RNAs (ncRNAs) [19, 20]. NcRNAs represent a subclass of RNAs that are transcribed but not translated with different lengths, structures, and functions. Interestingly, their interactions with other cellular RNAs and/ or proteins contribute to the creation of an intricate network capable of shaping cellular phenotypes through the regulation of both cellular transcription and translation, so that ncRNAs act as epigenetic regulators [21] which impact on cell proliferation, differentiation, cell death and metabolism, especially in cancer [22–25]. Long Non-Coding RNA (lncRNAs), a group of ncRNA longer than 200 nucleotides, are already known to play important roles in skin homeostasis, such as for example the Anti-Differentiation ncRNA (ANCR) and the Terminal-Differentiation Induced ncRNA (TINCR), which can regulate epidermal proliferation and differentiation, respectively [26, 27]. Different lncRNAs are involved in epidermal inflammatory response and in psoriasis insurgence, such as XIST, MEG3, FABP5B3, PRINS, and KLDHC7B-DT [28–30].

Ultra-conserved lncRNAs (T-UCR) represent a peculiar class of lncRNAs containing Ultra-Conserved genomic regions (UCR) within the transcript among mouse, rat, and human genomes, and are transcribed from almost 500 genomic loci containing UCRs that span in length from 200 to 779 bases [31–33]. Previously, we have demonstrated the importance of a lncRNA, namely uc.291, in inducing the transcription of keratinocyte differentiation genes located in the Epidermal Differentiation Complex (EDC), via interaction with ACTL6A [34]. In fact, during differentiation of keratinocytes, uc.291 can dislodge the binding of ACTL6A on EDC locus, allowing the release of chromatin by the SWI / SNF complex and determining the activation of differentiation genes located in the EDC locus, including loricrin (*LOR*) and filaggrin (*FLG*). Furthermore, dysregulation of uc.291-ACTL6A-SWI/SNF complex and consequent loss of differentiation program was also observed also in cutaneous tumours (basal cell carcinoma and squamous cell carcinoma) [35].

Given that impaired differentiation of keratinocytes occurs in psoriasis, we decided to focus our attention on the involvement of uc.291 and its interactor ACTL6a in this patology.

## Materials and methods

Seven patients with mild to severe chronic plaque psoriasis (Psoriasis area and severity index: 4–45) were included in this study. Biopsies were taken from skin plaques in both lesional and non-lesional areas (3 cm from the developing plaque), all from the same psoriatic patient. In parallel, skin biopsies were also taken from 9 healthy volunteers undergoing plastic surgery. Biopsies were cut in half and used for both RT-PCR and immunohistochemical studies. This study was approved by the Ethics Committee of the IDI-IRCCS Hospital, Rome (registration number: 475/1/2016) and carried out according to the Declaration of Helsinki. Informed consent was signed by all study subjects.

### RNA extraction, reverse transcription, and quantitative real-time PCR analysis

Total RNA was extracted from skin biopsies using the RNeasy Lipid Tissue Mini Kit (Qiagen, Hilden, Germany) and retrotranscribed using a SuperScript™ IV VILO™ Master Mix (thermo Fisher Scientific) according to the manufacturer’s protocol. Real-time PCR was performed using SYBR™ Green Master Mix (Thermo Scientific, Applied Biosystems). The primers used are listed in Additional file 1: Table 1. Expression of each gene was defined by the threshold cycle (Ct), and relative expression levels were calculated using the 2^−ΔΔCt^ method after normalisation with reference to expression of TBP as a housekeeping gene.

### Cell culture and cell treatment

Human epidermal keratinocytes, neonatal (HEKn), were purchased from ThermoFisher Scientific (Gibco ™) and cultured in EpiLife ™ medium (ThermoFisher scientific, Gibco TM) supplemented with Human Keratinocyte Growth Supplement (HKGS) (ThermoFisher scientific, Gibco™) [36]. HEKn were differentiated with 1.2 mM CaCl_2_ added to culture medium and collected at the following time points: 0 and 3 days. To induce the model of in vitro psoriasis, interleukin 22 (IL-22) (50 ng/µl, R&D systems) was added for 24 h to the differentiated cell culture medium. Then, untreated and treated cells were collected, and the cell pellet was processed.

### Immunohistochemical staining

Immunohistochemical staining for ACTL6A, Filaggrin, and Loricrin was performed using an anti-ACTL6A antibody (Cat. No. 76682, Cell Signalling), anti-Filaggrin antibody (Cat. No. PRB-417P, BioLegend) and anti-Loricrin antibody (Cat. No. PRB-145P, Covance) following the manufacturer’s instructions. For staining, sections were dewaxed and rehydrated and incubated to block endogenous peroxidases in a 0.03% solution of hydrogen peroxide in methanol [37]. Antigen retrieval was then performed by boiling the sample in 0.01 M citrate buffer pH 6.0 for 15 min in a 96 °C water bath. The slides were incubated with an anti-ACTL6A antibody (1: 400), a -Filaggrin antibody (1:200) or a -Loricrin antibody (1:1000) for 1 h at room temperature. Signals were detected using an UltraTek HRP antipolyvalent DAB staining system (ScyTek, Logan, UT, USA), and the slides were then counterstained with haematoxylin, dehydrated, and mounted. The slides were scanned using a DMI6 microscope, Leica microsystems.

### Bioinformatic analysis

Normalised values for the expression of *ACTL6A*, filaggrin, and loricrin RNA in healthy, psoriatic skin samples and IL-22 treated keratinocytes were obtained from the NCBI GEO portal. Normal, nonlesional, and lesional skin accession number: GSE13355; not treated: GSE7216.

### Statistical analysis

All statistical analyses were performed with GraphPad Prism 8.0 software (San Diego, CA, USA). For the analysis of the gene array data, the significance level (*p*) was calculated using Welch’s t test of unequal variances. Values of *p* < 0.05 were considered significant.

## Results

### uc.291 and *ACTL6A* expression is down- and up- regulated in lesional psoriasis, respectively

**Fig. 1:**
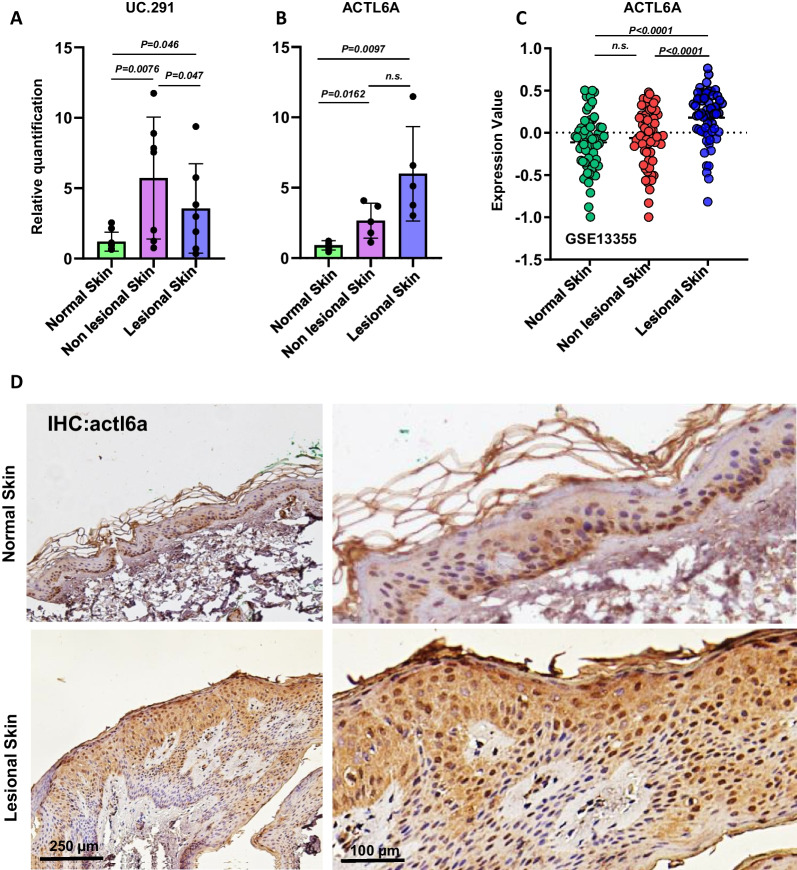
Expression of uc.291 and ACTL6A in normal epidermis and psoriatic non-lesional and lesional skin. **A** RT-qPCR analysis of uc.291 was performed on human normal skin (n = 9), and non-lesional (n = 7) and lesional (n = 7) areas in the skin of psoriatic patients. **B** ACTL6A mRNA expression level was performed by RT-qPCR on normal human skin (n = 5), non-lesional skin (n = 5), and lesional skin (n = 5). **C** Bioinformatic analysis of *ACTL6A* expression on normal human skin (n = 64) and from non-lesional (n = 54) and lesional (n = 54) areas of skin psoriatic patients (GSE13355). **D** Immunohistochemistry of ACTL6A on normal human skin (n = 3) and human skin affected by psoriasis (n = 3). Scale bar, 250 μm (left panels) and 100 μm (right panels). A representative experiment of three is shown. For all experiments and datasets analysis of data sets, the *p*-value was obtained using a t-test student; *p* < 0.05; n.s. not significant. Replicae are biological replicates

The contribution of uc.291 to the regulation of human keratinocytes differentiation by the interaction with ACTL6A protein has already been shown [34], however, the implication of this molecular mechanism in skin disorders like psoriasis, where keratinocyte differentiation is one of the events that are mainly affected, has not been elucidated yet. Therefore, we collected and analysed skin biopsies obtained from 9 healthy donors and from 7 patients with mild to severe chronic plaque psoriasis. Biopsies were taken from skin plaques of patients with psoriasis in both lesional and non-lesional areas, all from the same patient. Quantitative real-time PCR (RT-qPCR) analysis reveals that uc-291 expression is significantly up-regulated in nonlesional psoriatic skin samples compared to healthy skin, while resulting significantly down-modulated in the lesional counterparts (Fig. 1A**)**. On the contrary, *ACTL6A* expression is slightly but significantly overexpressed in nonlesional psoriatic skin compared to healthy skin, and even more in the lesional skin counterpart (Fig. 1B**).** To further confirm these data, we analysed a publicly available GEO data set (GSE13355) containing 64 human normal skin, 54 psoriatic non-lesional skin and 54 counterparts of lesional skin biopsies. ACTL6A expression value is significantly increased in the lesional skin compared to the non-lesional one, while there are non-significant differences between the normal and non-lesional skin **(**Fig. 1C**).** Furthermore, at the protein level, detected by immunohistochemistry, ACTL6A is also increased in psoriatic lesional skin compared to the normal skin (Fig. 1D). Taken together, these data suggest that in psoriatic lesional skin, the down regulation of uc.291 could account for impaired keratinocyte differentiation, mediated by ACTL6A.

### The expression of epidermal differentiation complex genes, filaggrin (*FLG*) and loricrin (*LOR*)*,* is affected in psoriasis

**Fig. 2:**
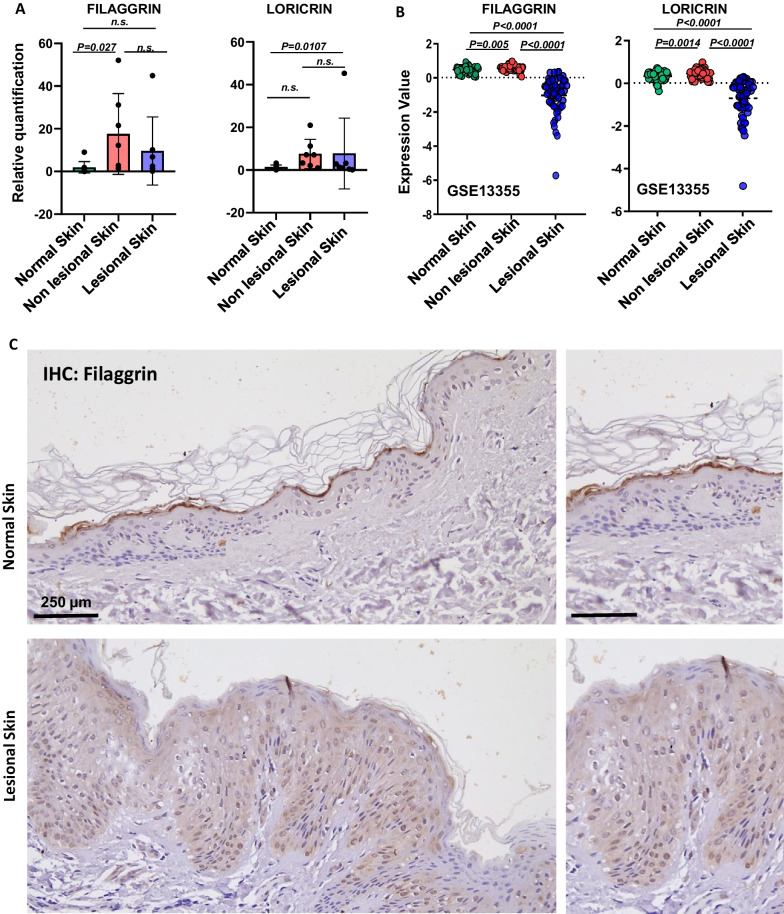
Expression of differentiation genes filaggrin and loricrin in normal epidermis and psoriatic non-lesional lesional skin. **A** Expression of *FLG* and *LOR* at the mRNA level. Relative quantification on normal human skin (n = 9), non-lesional (n = 7), and lesional skin (n = 7) was obtained by RT-qPCR. **B** Bioinformatics analysis of *FLG* and *LOR* expression in normal human skin (n = 64) and from non-lesional (n = 54) and lesional (n = 54) areas of skin psoriatic patients (GSE13355). **C** Immunohistochemistry of Filaggrin in normal human skin (n = 3) and human skin affected by psoriasis (n = 3). Scale bar, 250 μm (left panels) and 100 μm (right panels). A representative experiment of three is shown. For all experiments and datasets analysis of data sets, the *p*-value was obtained using a t-test student; *p* < 0.05; n.s. not significant. Replicae are biological replicates

Since lncRNA uc.291 can dislodge ACTL6A-chromatin binding on the EDC locus, allowing the activation of transcription of differentiation genes residing at the EDC locus, including filaggrin and loricrin, we decided to determine their expression in patients with psoriasis skin and in healthy donors. Using RT-qPCR, we found that the mRNA expression of both differentiation genes, *FLG* and *LOR*, increases in non-lesional skin as compared with normal biopsies, while it is slightly decreased in the lesional counterparts (Fig. 2A). Then we examined publicly available GEO datasets (GSE13355) and showed that the expression value of *FLG* as well as *LOR* is significantly decreased in psoriatic lesional skin from patients compared to healthy skin samples from donors (Fig. 2B). Furthermore, immunohistochemical staining of normal and psoriatic lesional skin biopsies reveals that Filaggrin expression is drastically reduced at the protein level in psoriasis (Fig. 2C). These results confirm that during psoriasis, skin differentiation and expression of some of its mediator genes (*FLG* and *LOR*) are primarily affected.

### The expression of uc.291, ACTL6A and EDC genes is impaired in *in-vitro* psoriasis-like model

**Fig. 3:**
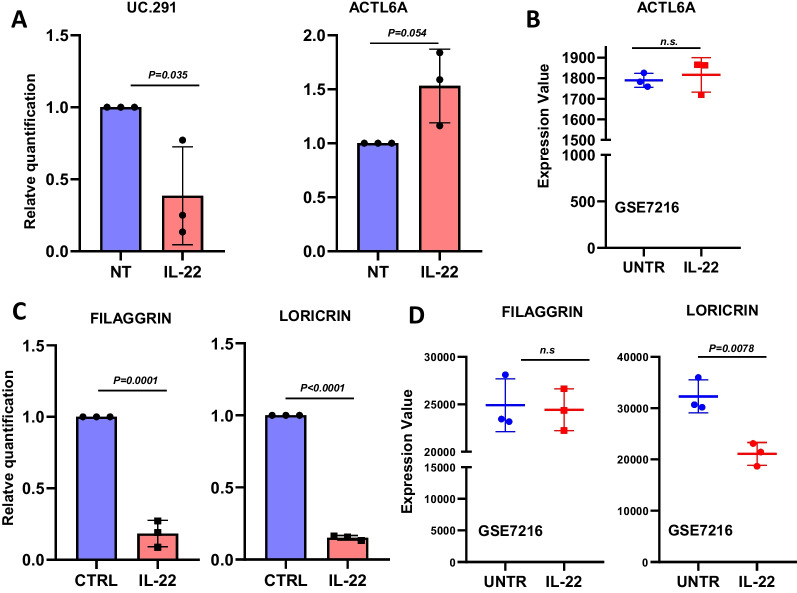
uc.291, ACTL6A, *FLG* and *LOR* expression in an in vitro psoriasis-like model. **A** Analysis of uc.291 and *ACTL6A* in human differentiated keratinocytes not treated (NT) and treated with IL-22 was determined by RT-qPCR (n = 3). **B** Bioinformatics analysis of *ACTL6A* in untreated human epidermal keratinocytes (UNT) (n = 3) and treated with IL-22 (n = 3) (GSE7216). **C** Analysis of *FLG* and *LOR* in human differentiated keratinocytes not treated (NT) and treated with IL-22, determined by RT-qPCR (n = 3). The *p*-value was obtained using t-test student; *p* < 0.05; n.s. not significant. **D** Bioinformatic analysis of *FLG* and *LOR* on Untreated (UNT) (n = 3) and IL-22 treated human epidermal keratinocytes (n = 3) (GSE7216). For all experiments and datasets analysis of data sets, the *p*-value was obtained using a t-test student; *p* < 0.05; n.s. not significant. Replicae are biological replicates

**Fig. 4:**
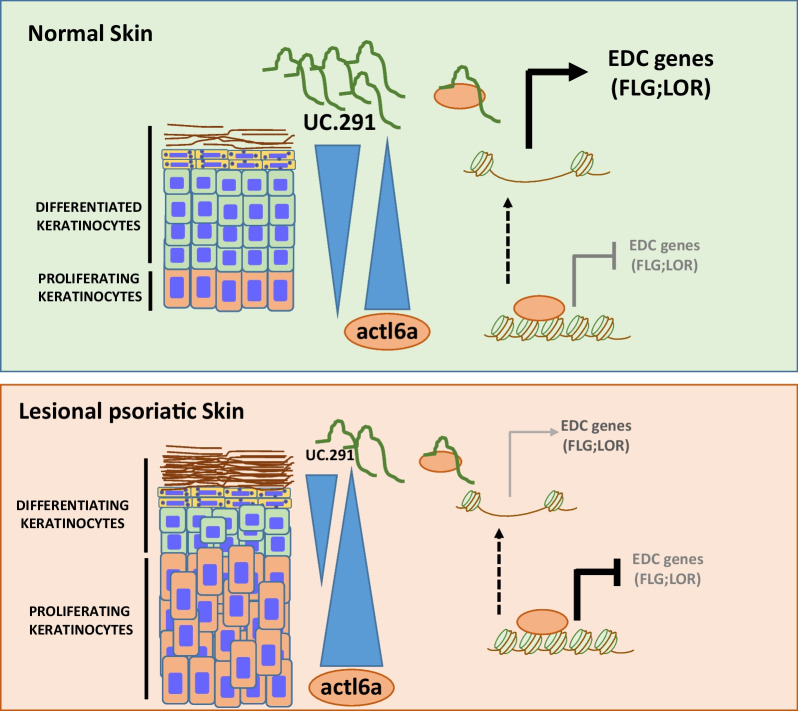
Model of the function of uc.291/ACTL6A in normal and psoriasis lesional skin. **A** In the basal layer (proliferating cells) of normal skin, ACTL6A is required for the inhibition of the expression of the EDC (epidermal differentiation complex) genes. Expression of uc.291 during differentiation of keratinocytes is important to allow the expression of EDC genes by binding and inhibiting ACTL6A. On psoriasis lesional skin (bottom panel), the expression of ACTL6A increases while uc.291 is reduced. As a consequence, the proliferating layer is thicker than in healthy skin and the differentiation process is affected

Among all the inflammatory stimuli responsible for the development of psoriasis, interleukin 22 (IL-22) can induce an imbalanced keratinocytes proliferation and differentiation[11]. To support our hypothesis on the involvement of uc.291 and its competitor ACTL6A in modulating keratinocyte differentiation, we decided to establish an in vitro model of psoriasis by treating differentiated cells with IL-22. Relative quantification of uc.291 and ACTL6A expression shows that they are significantly down- and up-regulated, respectively, in IL-22 treated keratinocytes compared to non-treated ones. (Fig. 3A). The expression value was also evaluated in the publicly available GEO dataset GSE7216, where 3 biological replicates of human keratinocytes treated with IL-22 versus 3 untreated counterparts were analysed. We showed that ACTL6A expression is slightly, but not statistically significantly, increased in IL-22 treated keratinocytes (Fig. 3B). Finally, to demonstrate that keratinocyte differentiation is impaired by deficiencies in filaggrin and loricrin, a RT-qPCR was used to measure their mRNA expression. The relative quantification of *FLG* and *LOR* expression shows that they are significantly reduced after IL-22 treatment compared to untreated cells (Fig. 3C). Furthermore, the analysis of the GSE7216 dataset shows that *LOR* mRNA expression is significantly down-regulated in IL-22 treated keratinocytes, while there is no change in *FLG* mRNA level between treated and untreated cells. In general, these results confirmed that in a model similar to psoriasis, induced by cytokines, down-regulation of uc.291 correlates to an impaired expression of *FLG* and *LOR*. This may be due to the overexpression of ACTL6A and to its permanence on the chromatin of the EDC locus due to uc.291 down-regulation, resulting in inhibition of the transcription of differentiation genes (Fig. 4**)**.

## Discussion

Physiological keratinocyte proliferation, differentiation, and stratification are fundamental for normal skin homeostasis and to maintain its protective role against external stimuli[1, 38] The balance between proliferation and differentiation in the epidermis is strictly regulated; however, different environmental and genetical issues can occur that cause cellular and molecular alterations responsible for different skin pathologies. Psoriasis is a chronic and inflammatory skin pathology, triggered by an unbalanced ratio between the proliferation and differentiation of human keratinocytes caused by hyperactivation and altered infiltration of inflammatory cells at dermis, in the sites of skin lesions[15]. Moreover, psoriasis is one of the most common health problems worldwide, and the effects on patient lives can be severe and also affect psychological health[11, 39]. Since psoriasis is an inflammatory disease, the risk that patients with severe forms may also develop a malignant skin disease or other systemic diseases is very high. In particular, non-melanoma skin cancers and lymphoproliferative malignant diseases are among the most common forms of cancer that can be associated with long-term severe psoriasis[40]. Given that human psoriasis is mainly caused by the alteration of keratinocyte proliferation and differentiation programmes, we decided to further understand the role of lnc-RNA uc-291, its molecular interactor ACTL6A, and the epidermal differentiation genes *FLG* and *LOR* in this disorder, considering that the involvement of this molecular axis has just been elucidated in physiological differentiation conditions[34]. This class of non-coding RNA is involved in different molecular processes due to their multifaceted functions and a dysregulation of their expression is often responsible for abnormal cellular homeostasis and for the insurgence and/or the maintenance of several pathologies, including cancer [41–44]. This is true for example for melanoma cancer, in which lncRNA alteration is responsible for cancer progression and malignant behavior of cells [45, 46]. In fact, taken into account their regulatory functions, lncRNAs represent good candidates for cancer diagnosis, prognosis, and treatment [47] and in particular in melanoma, the chemical inhibitor of the lncRNA LINC01212 has already been used as a treatment[48]. Here, we first demonstrated that uc.291 is down-regulated in lesional psoriatic skin compared to the nonlesional, while *ACTL6A* is upregulated, also at protein level, as shown by immunohistochemistry. Our data are in line with the results obtained after the analysis of the publicly available GEO dataset (GSE13355), which confirmed ACTL6A overexpression in lesional skin samples compared to nonlesional and normal ones (Fig. 1). Then, we demonstrated that the expression of EDC genes *FLG* and *LOR* is slightly decreased in lesional psoriasis compared to non-lesional one and significantly decreased in lesional samples of the GSE13355 dataset. In addition, immunohistochemistry shows a strong reduction in Filaggrin expression in the upper differentiated layer of human psoriatic skin compared to the normal counterpart (Fig. 2). Finally, we used an in vitro model of psoriasis in which human differentiated keratinocytes are treated with interleukin 22 (IL-22). This treatment induced a decrease of uc.291 expression but an increase in ACTL6A mRNA level and a notable decrease of *FLG* and *LOR* expression. These results are partially confirmed by the bioinformatical analysis of the publicly available GEO dataset GSE7216 (Fig. 3). Therefore, we propose a molecular model in which the decrease of uc.291 and increase of ACTL6A trigger EDC genes (*FLG* and *LOR)* expression in human lesional psoriatic skin (Fig. 4). Collectively, these data highlight the role of uc.291 not only in skin physiology under normal conditions but also in psoriasis, making it an interesting candidate for further studies and future potential drug development for hyperproliferative skin disorders.

### Supplementary Information

**Additional file 1.** Table 1.

## Data Availability

The authors declare that the data supporting the findings of this study are available in the paper and its supplementary information files.
