# Supplementary material for: Involvement of transcribed lncRNA uc.291 in hyperproliferative skin disorders
Source: Biol Direct. 2023 Dec 1;18:82. doi: 10.1186/s13062-023-00435-0 (PMC10693168; doi:10.1186/s13062-023-00435-0)
Supplement: Supplementary file 1 — Additional file 1. Table 1. [file 13062_2023_435_MOESM1_ESM.docx]

**Supplementary table 1**

| **Gene name** | **Sequence 5’-3’** |
| --- | --- |
| T-UC291 FW | GCGTCAATGTTCATCTGTAATTC |
| T-UC291 REV | CTGTTCTCAGCCTGTGCCGAG |
| LOR FW | CTCTGTCTGCGGCTACTCTG |
| LOR REV | CACGAGGTCTGAGTGACCTG |
| FLG FW | CAATCAGGCACTCATCACAC |
| FLG REV | ACTGTTAGTGACCTGACTACC |
| TBP FW | TCAAACCCAGAATTGTTCTCCTTAT |
| TBP REV | CCTGAATCCCTTTAGAATAGGGTAGA |
| ACTL6A FW | TAATGCTCTGCGTGTTCCGA |
| ACTL6A REV | CGGTGCCTCTGACATGAGAA |
